# Threshold-based disease treatment approach modulates economic, conservation and evolutionary trade-offs in sea louse-salmon aquaculture system

**DOI:** 10.1007/s12080-025-00617-8

**Published:** 2025-06-06

**Authors:** Laurinne J. Balstad, Sean C. Godwin, Martin Krkošek, Mark A. Lewis, Marissa L. Baskett

**Affiliations:** 1 Department of Environmental Science and Policy, University of California, Davis, 2132 Wickson Hall, One Shields Avenue, Davis, 95616 CA USA; 2 Center for Population Biology, University of California, Davis, 2320 Storer Hall, One Shields Avenue, Davis, 95616 CA USA; 3 Bodega Marine Laboratory, University of California, Davis, 2099 Westshore Road, Bodega Bay, 94923 CA USA; 4 Department of Ecology and Evolutionary Biology, University of Toronto, 25 Willcocks Street, Toronto, M5S 3B2 ON Canada; 5 Department of Mathematics and Statistics and Department of Biology, University of Victoria, PO Box 1700 Station CSC, Victoria, V8W 2Y2 BC Canada

**Keywords:** Salmon aquaculture, *Lepeophtheirus salmonis*, Treatment resistance, Parasite refugia, Efficiency frontiers, Mathematical model

## Abstract

Mitigating negative downstream impacts of parasitic disease in aquaculture settings entails tradeoffs: reducing parasite loads has economic and conservation benefits, but treatment is often expensive and frequent treatment can lead to resistance evolution. Options for mitigating these potential trade-offs depend on the management context. For example, in the sea louse-salmon system, managers use discrete treatment applications to control louse burdens, applying treatment when parasite burdens exceed a target threshold. To analyze the effect of a threshold-based control of disease treatment on economic, conservation, and evolutionary outcomes, we incorporate discrete treatment into a dynamical model of sea louse-salmon systems with disease spillover to wild populations. The model follows both salmon hosts and sea lice through domestic, wild, and migratory populations, with treatment occurring when sea lice exceed a target threshold. Our model shows that simultaneous economic and conservation win-wins are possible: there are treatment threshold choices that lead to relatively high wild juvenile salmon population sizes and relatively low economic losses, especially when treatment is very effective or treatment is cheap. However, positive evolutionary outcomes are harder to capture and occur most often when treatment efficacy is low and the treatment threshold is either near zero or very high. Expanding the management toolbox beyond choices of treatment threshold and treatment efficacy could help managers better capture positive economic, evolutionary and conservation outcomes in the system.

## Introduction

Aquaculture production systems often stock animals at higher densities than their wild counterparts, which can increase disease burdens and lead to negative economic and conservation outcomes (Krkošek 2010a). Economically, hosts with high disease burdens can have decreased value, reducing producer profits (Lafferty et al. 2015). With regards to conservation, the exchange of parasites between domestic and sympatric, wild populations can lead to population declines in some systems (Bouwmeester et al. 2021). To alleviate these consequences, domestic hosts are often heavily treated to reduce disease. However, this intensive treatment is economically costly and can create strong selection pressure for treatment resistance among parasites (Aaen et al. 2015; Coates 2023), eroding producers’ ability to manage the disease over time. Management choices such as the degree of treatment application are central to balancing the consequences of disease across economic, conservation, and evolutionary outcomes (Jansen et al. 2012; Groner et al. 2016; Coates et al. 2023).

These disease effects and management considerations are exemplified in the sea louse-salmon aquaculture system. Economically, sea lice cause upwards of US$436 million in direct and indirect costs to the Norwegian salmon aquaculture industry alone (Abolofia et al. 2017); more conservative estimates put the direct cost of sea lice control between €0.1 and 0.19 per kilogram of salmon produced (Costello 2009). With regards to conservation, sea lice can lead to depressed sympatric wild salmon populations when they spill back onto juvenile wild salmon (Krkošek et al. 2011; Peacock et al. 2013; Kristoffersen et al. 2018). Widespread chemical treatment has led to evolution of treatment resistance across the globe (Lees et al. 2008; Aaen et al. 2015; Coates et al. 2021; Fjørtoft et al. 2019, 2020; Quiñones et al. 2019). Even in British Columbia (BC), Canada, where resistance was once rare, resistance to chemical treatments is becoming more common (Godwin et al. 2022). Resistance to chemical treatments has led aquaculture to use more frequent chemical treatment or move to from chemical treatments to different, more expensive, and/or less effective treatments, such as hydrogen peroxide baths or mechanical delousing (Overton et al. 2019; Barrett et al. 2020; Boerlage et al. 2024).

Analysis of the sea louse-salmon system indicates the possible benefits and costs of alternative management approaches (Groner et al. 2016; Coates 2023). Models have demonstrated that some treatment of sea lice can be economically optimal for producers: this balances the cost of treatment with the loss of salmon host value associated with sea lice (Murray 2011). Empirical and modeling work shows that decreases in domestic salmon parasite burden through heavy treatment and decreases in spillback between domestic and juvenile wild salmon (e.g., by carefully timing treatment) helps reduce the negative effects of salmon aquaculture to wild salmon (Krkošek 2010b; Krkošek et al. 2011). Past modeling has highlighted two treatment application strategies that help slow the selection of treatment resistance: the low treatment strategy (Murray 2011; Stratonovitch et al. 2014) and the high-dose refuge strategy (Ives and Andow 2002; Ashander 2010; McEwan et al. 2015; Kreitzman et al. 2017; Bateman et al. 2020). The low treatment strategy minimizes selection and, given a high economic cost associated with treatment, tends to lead to lower economic losses (Murray 2011). The high-dose refuge strategy uses intensive treatment in the production environment to reduce parasite burdens, coupled with large subsidies of wild (treatment susceptible) parasites; in other words, gene flow outweighs local selection (Slatkin 1987; Lenormand 2002). To maintain a successful high-dose refuge strategy, wild populations must be large, highlighting the “evosystem” service of wild parasite and wild salmon host populations (Ashander 2010; Kreitzman et al. 2017; Bateman et al. 2020).

While past studies have shown the potential effectiveness of the low-treatment and high-dose refuge strategies using continuous treatment approaches (Murray 2011; Bateman et al. 2020), in reality, regulation and economic concerns often limit farms to discrete, pulsed treatment application only when parasite burdens are high (Krkošek 2010b; Jeong et al. 2023). In addition to raising the management question of treatment threshold, choices for discrete treatment regarding treatment type (e.g., chemical compared to mechanical treatment) and effort (e.g., chemical concentration) can control their efficacy (Overton et al. 2019; Barrett et al. 2020; Boerlage et al. 2024). Several studies have demonstrated the efficacy of both the low-treatment and high-dose refuge strategies using discrete treatment applications in agriculture and aquaculture pest control (Stratonovitch et al. 2014; McEwan et al. 2015; Coates et al. 2023), but the combined effect of treatment threshold and efficacy choices on resistance evolution, conservation outcomes and farm economics, and their potential trade-offs with each other, has not been studied. Adjustments to treatment threshold and treatment efficacy might modulate evolutionary outcomes by altering selection strength and gene flow, conservation outcomes by controlling the cap on domestic parasite burden that can spill over, and economic outcomes by controlling treatment frequency. Therefore, evaluating the effect of the treatment threshold value on all three outcomes can inform decisions targeted at achieving multiple outcomes with realism in the focal decision.

Here, we use a dynamical model with discrete, threshold-based treatment application to explore evolutionary, conservation, and economic outcomes in the sea louse-salmon system. We simulate populations of salmon and louse across three environments (domestic, coastal marine, and oceanic marine), paralleling the structure of Bateman et al. (2020). We decompose discrete treatment into treatment efficacy (i.e., the proportion of lice that die immediately following treatment) and treatment threshold. We consider the effect of different discrete treatment strategies and disease management techniques on (1) on-farm resistance evolution, (2) wild salmon population size, and (3) economic losses from disease burden and treatment cost.

**Fig. 1:**
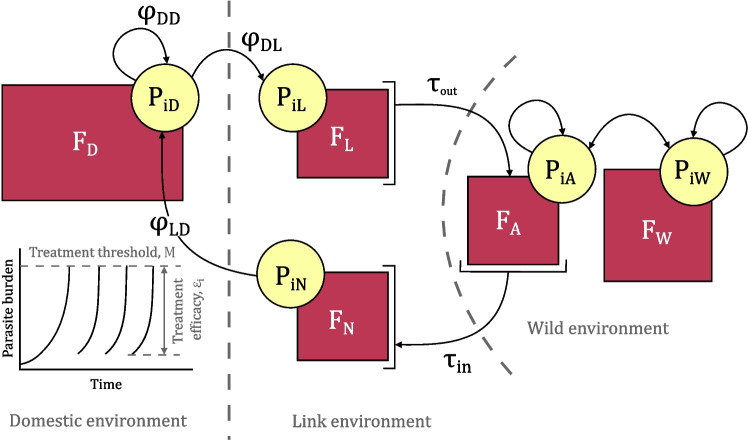
Model schematic. Adult lice (light yellow circles; $P_{ik}$, where $i \in (S,R)$ gives lice type and *k* gives the host type) are attached to hosts (dark pink squares; $F_k$) across each of the three environments (domestic, *D*; link, *L*; wild, *W*). Lice move from the link environment to the farm environment via spillover ($\phi _{DL}$) from $F_N$ spawning adult salmon hosts; lice move from the domestic to the link environment via spillback ($\phi _{LD}$) onto $F_L$ juvenile salmon host. Lice move from the link environment to the wild environment ($\tau _{out}$) and back via host migration ($\tau _{in}$). In addition to parasite movement between each environment, lice reproduce within each of the domestic and wild environments. In the domestic environment, treatment is applied when lice reach a pre-determined burden (*M*); treatment kills some proportion of the lice given by the treatment efficacy ($\epsilon _i$). Adapted from Bateman et al. (2020)

**Table 1:** State variable definitions

State variable	Meaning	Environment
$F_D$	Domestic salmon hosts, constant	Domestic environment
$F_J$	Juvenile salmon	Link environment
$F_A$	Adult migrating salmon	Wild environment
$F_W$	Wild salmon, constant	Wild environment
$F_N$	Spawning salmon	Link environment
$C_{ik}$	Free-living juvenile parasites, type *i*, associated with type *k* hosts	–
$P_{ik}$	Attached adult parasites, type *i*, associated with type *k* hosts	–

## Methods

### Model overview

Our model considers three environments following Bateman et al. (2020): a domestic (production) environment (*D*), a link environment (with juvenile salmon, *J* and spawning salmon, *N* hosts), and a wild environment (with adult salmon, *A*, and additional wild salmon *W*; Fig. 1). For each environment *k*, we simulate relevant host populations ($F_k$) and two sets of attached, adult louse types, a treatment-susceptible type ($P_{Sk}$) and a treatment-resistant type ($P_{Rk}$; Table 1). Hosts in the domestic and wild environments are confined to their respective environments, while hosts in the link environment migrate between the link environment and wild environment. This mimics the salmon system where juvenile and spawning salmon migrate through coastal marine environments past domestic salmon farms towards the ocean, where they mix with other salmon populations which have not necessarily been near domestic salmon. Louse movement occurs when lice spill over and spill back between link and domestic environments, and when lice are attached to migrating salmon between the link and wild environments.

Treatment is only applied in the domestic environment at times **T**, when the parasite burden reaches the treatment threshold in the domestic environment. We define the treatment strategy as the combination of two factors, the threshold that triggers treatment (*M*) and the treatment efficacy ($\epsilon _i$). Given this broad definition of treatment, our model can describe both chemical and non-chemical treatments that meet the following criteria: (1) treatment is applied at a pre-specified threshold, (2) treatment has some given efficacy, and (3) lice can evolve resistance to the treatment (Coates et al. 2021). In our model, resistant lice have the evolutionary benefit of reduced treatment mortality (i.e., decreased treatment efficacy, $\epsilon _R = \gamma \epsilon _S$, where $\gamma$ is the relative selective efficacy between louse types and $0< \gamma < 1$), which comes at the evolutionary cost of reduced fecundity ($\frac{\lambda _R}{\lambda _S} < 1$).

### Model construction

We derive the model from the assumptions of the standard macroparasite models by May and Anderson (1979) and Dobson (1985). We make the simplifying assumption that the two parasite types ($P_{Sk}, P_{Rk}$) are distributed following independent, identical, Poisson distributions in each environment. Using a pulse-impulsive or semi-discrete model structure (Mailleret and Lemesle 2009), we follow all dynamics except treatment over continuous time (domestic host stocking and harvest, wild host reproduction, mortality, and migration, and sea louse reproduction, attachment, detachment, and mortality as well as migration with hosts) and then apply treatment in discrete pulses when the parasite burden in the domestic environment meets the treatment threshold, at times **T**. All parameters, their meanings, and values used in simulations are given in Table 2.

**Table 2:** Parameter values and definitions. System-specific parameter values are used in Fig. 7 and generic values used elsewhere with modifications as indicated

Parameter	Meaning	Generic values	System-specific values (source)
$\lambda _S$	Susceptible parasite reproduction	1 larvae $\cdot$ louse$^{-1}$ time$^{-1}$	6.35 larvae $\cdot$ louse$^{-1}$ day$^{-1}$ (Frazer et al. 2012)
$\lambda _R$	Resistant parasite reproduction	$0.95*\lambda _S$	$0.95*\lambda _S$
$\nu _S, \nu _R$	Free-living parasite mortality	1 time$^{-1}$	0.2 day$^{-1}$ (Frazer et al. 2012)
$\mu _S, \mu _R$	Adult parasite mortality	0.1 time$^{-1}$	0.017 day$^{-1}$ (Frazer et al. 2012)
$\rho _D$	Density-dependent parasite mortality, domestic environment	0.15 salmon $\cdot$ louse$^{-1}$ time$^{-1}$	0.01 salmon $\cdot$ louse$^{-1}$ day$^{-1}$ (adjusted for realistic parasite burden)
$\rho _W$	Density-dependent parasite mortality, wild environment	0.15 salmon $\cdot$ louse$^{-1}$ time$^{-1}$	0.001 salmon $\cdot$ louse$^{-1}$ day$^{-1}$ (adjusted for realistic parasite burden)
$\alpha$	Parasite-induced host mortality, link environment	0.4 salmon $\cdot$ louse$^{-1}$ time$^{-1}$	0.02 salmon $\cdot$ louse$^{-1}$ day$^{-1}$ (Peacock et al. 2014)
*M*	Treatment threshold	Varies, 0.01–1.31 lice	Varies, 0.01–60.01 lice
$\epsilon _S$	Treatment efficacy, susceptible lice (unitless proportion)	Varies, 0.3–0.9	Varies, 0.3–0.9
$\gamma$	Relative selective efficacy between louse types, i.e., evolutionary resistance benefit (unitless proportion)	0.05	0.5
$\epsilon _R$	Treatment efficacy, resistant lice	$\gamma \epsilon _S$	$\gamma \epsilon _S$
$\phi _{DD}$	Proportion of lice remaining in domestic environment (unitless proportion)	0.9	0.9
$\phi _{DL}, \phi _{LD}$	Proportion of lice moving between domestic and link environments (unitless proportion)	$1-\phi _{DD} = 0.05$	$1-\phi _{DD}= 0.1$
$\phi _{WW}$	Proportion of lice remaining in the wild environment (unitless proportion)	1	1
$\beta _{D}$	Transmission rate, domestic environment	1 salmon$^{-1}$time$^{-1}$	$4.54*10^{-10}$ salmon$^{-1}$day$^{-1}$ (Frazer et al. 2012)
$\beta _{L}$	Transmission rate, link environment	$0.75*\beta _D$	$0.75*\beta _D$
$\beta _{W}$	Transmission rate, link environment	$0.25*\beta _D$	$0.25*\beta _D$
$Z_D$	Harvest rate, domestic environment	0.1 time$^{-1}$	0.002 day$^{-1}$ (Bateman et al. 2020)
$\zeta _J$	Host mortality, $F_J$ hosts	0.1 time$^{-1}$	0.01 day$^{-1}$ (Bateman et al. 2020)
$\zeta _N$	Host mortality, $F_N$ hosts	0.5 time$^{-1}$	0.01 day$^{-1}$ (Bateman et al. 2020)
$\zeta _W$	Host mortality, $F_W$ hosts	0.1 time$^{-1}$	0.005 day$^{-1}$
$F_D^*$	Number of domestic hosts, constant	1 salmon	$6*10^6$ salmon (Frazer et al. 2012)
$H_D$	Restocking rate, $F_D$ hosts	0.1 time$^{-1}$	0.01 day$^{-1}$
$\eta _{N1}$	Maximum reproduction, $F_N$ hosts	1.5 salmon $\cdot$ time$^{-1}$	$3*10^2$ salmon $\cdot$ day$^{-1}$
$\eta _{N2}$	Strength of density-dependent reproduction, $F_N$ hosts	1 salmon	$2*10^2$ salmon
$\eta _W$	Reproduction, $F_W$ hosts	0.1 time$^{-1}$	0.005 day$^{-1}$
$\tau _{out}$	Out-migration rate, rate $F_L$ hosts leave link environment	0.2 time$^{-1}$	0.01 day$^{-1}$ (Peacock et al. 2014)
$\tau _{in}$	In-migration rate, rate $F_A$ hosts enter link environment	0.1 time$^{-1}$	0.002 day$^{-1}$ (Bateman et al. 2020)
$F_W^*$	Number of wild hosts, constant	$5*F_D$	$20*F_D$ (Bateman et al. 2020)
$V_T$	Per treatment cost	0.015 (low cost), 0.045 (high cost) monetary unit	2.5 monetary unit
$V_P$	Cost of not treating, per louse	1 monetary unit	0.1 monetary unit

In the domestic environment, *D*, salmon hosts ($F_D$) are stocked at rate $H_D$ and harvested at rate $Z_D$:

1 $$\begin{aligned} \begin{array}{l c l l} \displaystyle \frac{\textrm{d}F_D}{\textrm{d}t}= &  H_D F_D - Z_D F_D ,&t \not \in {\textbf {T}}. \end{array} \end{aligned}$$

Attached adult lice ($P_{iD}$) in the domestic environment produce free-living juvenile lice at rate $\lambda _i$, where $i = S, R$. We consider spillover and spillback of lice by assuming that some fraction of the free-living juvenile lice are transferred between domestic and link environments. Specifically, $\phi _{DD} \lambda _i P_{iD}$ free-living lice of type $i \in (S, R)$ are produced and remain in the domestic environment and $\phi _{DL} \lambda _i P_{iS}$ free-living lice are produced in the link environment and spill over into the domestic environment. Free-living juvenile lice ($C_{iD}$) die at rate $\nu _i$ and attach to salmon at rate $\beta _D F_D$. The change in free-living juvenile lice is given by:

2 $$\begin{aligned} \displaystyle \frac{\textrm{d}C_{iD}}{\textrm{d}t}= &  \lambda _i \left( \phi _{DD} P_{iD} + \phi _{DL} P_{iS} \right) - \nu _{iD} C_{iD}\nonumber \\ &  - \beta _D F_D C_{iD}, \quad t \not \in {\textbf {T}}. \end{aligned}$$

Once attached to salmon, adult lice die from natural mortality at rate $\mu _i$, salmon harvest, and density-dependent competition for host resources, $\rho _D$. The adult lice in the domestic environment is described by:

3 $$\begin{aligned} \displaystyle \frac{\textrm{d}P_{iD}}{\textrm{d}t} = \beta _D F_D C_{iD} - \left( \mu _i + Z_D + \rho _D \frac{P_{iD} + P_{jD}}{F_D} \right) P_{iD}, \,\,\, t \not \in {\textbf {T}} \end{aligned}$$

for $i = S,R$ type lice and *j* is the other louse type. In the domestic environment, treatment occurs in discrete pulses. When the parasite burden reaches some triggering threshold, $\frac{P_{SD} + P_{RD}}{F_D} > M$, at times ${\textbf {T}}$, treatment is applied and a proportion of lice immediately dies, defined by the treatment efficacy $\epsilon _i$:

4 $$\begin{aligned} P_{SD}({\textbf {T}}^+)= &  \epsilon _S P_{SD}({\textbf {T}}^-) \nonumber \\ P_{RD}({\textbf {T}}^+)= &  \epsilon _R P_{RD}({\textbf {T}}^-) \end{aligned}$$

and all other state variables remain unmodified. Treatment only occurs in the domestic environment. This form of pulsed treatment reflects the regulation-required treatment common across many salmon aquaculture settings (Vormedal 2023), while the treatment efficacy reflects the imperfect nature of treatment in these systems (Overton et al. 2019; Barrett et al. 2020; Boerlage et al. 2024). The use of discrete treatment pulses allows us to consider two dimensions of selection: treatment threshold and treatment efficacy. Combined, these determine the number of times treatment is applied and the strength of selection for a specific treatment.

In the link environment, we model a salmon population with three life stages (out-migrating juvenile, $F_J$; adult, $F_A$; in-migrating spawning, $F_N$). Out-migrating juvenile salmon hosts die from both natural mortality at rate $\zeta _J$ and parasite-induced mortality at rate $\alpha$, proportional to parasite burden. Note that out-migrating juvenile hosts are the only hosts that die from parasite-induced mortality. In the sea louse-salmon system, juvenile wild salmon are most sensitive to louse burdens, while adult and domestic salmon typically experience only sublethal effects of parasites that likely do not influence their population dynamics (Peacock et al. 2019). Out-migrating juvenile hosts mature into adults and leave the link environment for the wild environment at rate $\tau _{out}$. Adult salmon hosts are well-mixed with wild salmon hosts in the wild environment and leave the wild environment to become in-migrating spawning hosts at rate $\tau _{in}$. In-migrating spawning salmon hosts produce juvenile salmon following density-dependent reproduction (where $\eta _{N1}$ gives the production rate and $\eta _{N2}$ gives the strength of density-dependence) and die at rate $\zeta _N$. Then, the population dynamics for the salmon population in the link environment are given by:

5 $$\begin{aligned} \left. \begin{array}{lcl} \displaystyle \frac{\textrm{d}F_J}{\textrm{d}t} & =& \frac{\eta _{N1} F_N}{\eta _{N2} + F_N} - (\zeta _J + \tau _{out}) F_S - \alpha (P_{iJ} + P_{jJ}) \\ \displaystyle \frac{\textrm{d}F_A}{\textrm{d}t} & =& \tau _{out} F_J- \tau _{in} F_A \\ \displaystyle \frac{\textrm{d}F_N}{\textrm{d}t} & =& \tau _{in} F_A - \zeta _N F_N \\ \end{array} \right. t \not \in {\textbf {T}}. \end{aligned}$$

We modelled two parasite populations in the link environment, parasites associated with out-migrating juvenile salmon hosts and parasites associated with in-migrating spawning salmon hosts; parasites associated with adult salmon hosts are well-mixed with parasites in the wild environment and tracked there. First, adult parasites attached to out-migrating juvenile salmon hosts do not reproduce: the only production for these parasites comes from spillback sourced from the domestic environment (proportion $1-\phi _{DD}$ of the domestic production $\lambda _{i}P_{iD}$, described above). Free-living juvenile parasites from the domestic environment die at rate $\nu _i$ and attach to juvenile salmon hosts at rate $\beta _L F_J$. Adult parasites attached to juvenile salmon hosts die from natural parasite mortality, die from host mortality, and leave the link environment at rate $\tau _{out}$ via host out-migration. When adult parasites kill juvenile hosts, with parasite-induced mortality rate $\alpha$, they do so in proportion to the parasite burden. The resulting host death will then kill any other parasites attached to the host; this results in non-linear parasite mortality due to virulence (derived in May and Anderson , 1979; Dobson , 1985). The parasites associated with out-migrating juvenile salmon hosts are:

6 $$\begin{aligned} \left. \begin{array}{lll} \displaystyle \frac{\textrm{d}C_{iJ}}{\textrm{d}t} & =& (1-\phi _{DD}) \lambda _i P_{iD} - \nu _i C_{iJ} -\beta _L F_J C_{iJ} \\ \displaystyle \frac{\textrm{d}P_{iJ}}{\textrm{d}t} & =& \beta _L F_J C_{iJ} - (\mu _i + \zeta _J + \tau _{out} + \alpha ) P_{iJ} \\ & & - \alpha \frac{P_{iJ} + P_{jJ}}{F_J} P_{iJ}\\ \end{array}\right. t \not \in {\textbf {T}} \end{aligned}$$

for $i = S,R$ type lice and *j* is the other louse type. Second, adult parasites associated with in-migrating spawning hosts reproduce at rate $\lambda _i$ to produce free-living juvenile parasites. Some fraction $\phi _{LL}$ of the free-living parasites remain in the link environment and the remainder spill over into the domestic environment. Free-living parasites die at rate $\nu _i$ and attach to in-migrating spawning hosts at rate $\beta _L F_S$. Additionally, adult parasites arrive in the link environment due to in-migration by adult hosts at rate $\tau _{in}$. Adult parasites attached to spawning hosts die from natural mortality and natural host death. Then, the parasites associated with spawning hosts are described by:

7 $$\begin{aligned} \left. \begin{array}{lcl} \displaystyle \frac{\textrm{d}C_{iN}}{\textrm{d}t} & =& \phi _{LL} \lambda _i P_{iN} - \nu _i C_{iN} -\beta _L F_S C_{iN}\\ \displaystyle \frac{\textrm{d}P_{iN}}{\textrm{d}t} & =& \beta _L F_N C_{iN} - (\mu _i + \zeta _N) P_{iN} + \tau _{in} \frac{F_A}{F_A+F_W} P_{iW}\\ \end{array} \right. \!\! t\! \not \in \! {\textbf {T}} \end{aligned}$$

for $i = S,R$ type lice and *j* is the other louse type.

Finally, we consider hosts in the wild environment. There are two types of hosts in the wild environment, adult salmon hosts ($F_A$), which have migrated from the link environment (Eq. 5) and wild hosts ($F_W$), which remain in the wild environment. Wild hosts are born at rate $\eta _W$ and die at rate $\zeta _W$:

8 $$\begin{aligned} \displaystyle \frac{\textrm{d}F_W}{\textrm{d}t} = \eta _W F_W - \zeta _W F_W, \qquad \quad t \not \in {\textbf {T}}. \end{aligned}$$

Adult parasites in the wild environment produce free-living parasites at rate $\lambda _i$. Free-living parasites die at rate $\nu _i$ and attach to both adult salmon and wild salmon hosts at rate $\beta _W (F_W + F_A)$. In addition to free-living parasites maturing into adult parasites, adult parasites can also arrive to and leave, this environment via migration of the link population. Attached adult parasites in the wild environment die from natural mortality at rate $\mu _i$, host death, and density-dependent competition for host resources, $\rho _W$. The lice in the wild environment is described by:

9 $$\begin{aligned} \left. \begin{array}{lcl} \displaystyle \frac{\textrm{d}C_{iW}}{\textrm{d}t} & =& \phi _{WW} \lambda _i P_{iW} - \nu _i C_{iW} - \beta _W (F_A + F_W) C_{iW}\\ \displaystyle \frac{\textrm{d}P_{iW}}{\textrm{d}t} & =& \beta _W (F_W + F_A) C_{iW} + \tau _{out} P_{iJ} \\ & & - \left( \zeta _W \frac{F_W}{F_W + F_A} + \mu _i + \tau _{in} \frac{F_A}{F_A + F_W} + \rho _W \frac{P_{iW} + P_{jW}}{F_A + F_W} \right) P_{iW} \\ \end{array} \right. t \not \in  {\textbf {T}}. \end{aligned}$$

for $i = S,R$ type lice and *j* is the other louse type.

### Model parameterization and simulations

Next, we make a set of steady-state assumptions to simplify the system: (1) free-living parasites ($C_{ik}$) are at equilibrium, i.e., their population dynamics are much faster than those of attached adult parasites and (2) domestic ($F_D^*$) and wild salmon hosts ($F_W^*$) are at equilibrium, $H_D = Z_D$ and $\eta _W = \zeta _W$. These assumptions result in the following dynamic equations we simulate:

10 $$\begin{aligned} \left. \begin{array}{lcl} \displaystyle \frac{\textrm{d}F_J}{\textrm{d}t} & =& \frac{\eta _{N1} F_N}{\eta _{N2} + F_N} - (\zeta _J + \tau _{out}) F_S - \alpha (P_{iJ} + P_{jJ}) \\ \displaystyle \frac{\textrm{d}F_A}{\textrm{d}t} & =& \tau _{out} F_J- \tau _{in} F_A \\ \displaystyle \frac{\textrm{d}F_N}{\textrm{d}t} & =& \tau _{in} F_A - \zeta _N F_N \\ \displaystyle \frac{\textrm{d}P_{iD}}{\textrm{d}t} & =& \beta _D F_D^* \left( \displaystyle \frac{\lambda _i \phi _{DD} P_{iD} + \lambda _i \phi _{DL} P_{iN}}{\nu _i + \beta _D F_D^*} \right) \\ & & - \left( \mu _i + Z_D + \rho _D \displaystyle \frac{P_{iD} + P_{jD}}{F_D^*} \right) P_{iD}\\ \displaystyle \frac{\textrm{d}P_{iJ}}{\textrm{d}t} & =& \beta _L F_J \left( \displaystyle \frac{\lambda _i (1-\phi _{DD}) P_{iD}}{\nu _i + \beta _L F_J} \right) \\ & & - (\mu _i + \zeta _J + \tau _{out} + \alpha ) P_{iJ}- \alpha \displaystyle \frac{P_{iJ} + P_{jJ}}{F_J} P_{iJ}\\ \displaystyle \frac{\textrm{d}P_{iW}}{\textrm{d}t} & =& \beta _W (F_W^* + F_A) \left( \displaystyle \frac{\lambda _i P_{iW}}{\nu _i + \beta _W(F_A + F_W^*)} \right) + \tau _{out} P_{iJ} \\ & & - \left( \zeta _W \frac{F_W^*}{F_W^* + F_A} + \mu _i + \tau _{in} \displaystyle \frac{F_A}{F_A + F_W^*} + \rho _W \displaystyle \frac{P_{iW} + P_{jW}}{F_A + F_W^*} \right) P_{iW} \\ \displaystyle \frac{\textrm{d}P_{iN}}{\textrm{d}t} & =& \beta _L F_N \left( \displaystyle \frac{\lambda _i \phi _{LL} P_{iN}}{\nu _i + \beta _L F_N} \right) - (\mu _i + \zeta _N) P_{iN} + \tau _{in} \displaystyle \frac{F_A}{F_A+F_W^*} P_{iN}\\ \end{array} \right. t \not \in {\textbf {T}} \end{aligned}$$

for $i = S,R$ type lice and *j* is the other louse type. As above, treatment occurs only in the domestic environment, when the parasite burden exceeds the pre-specified treatment threshold ($\frac{P_{SD} + P_{RD}}{F_D} > M$), given by times ${\textbf {T}}$. When treatment is applied, a proportion of lice immediately dies, defined by the treatment efficacy $\epsilon _i$:

11 $$\begin{aligned} P_{SD}({\textbf {T}}^+)= &  \epsilon _S P_{SD}({\textbf {T}}^-) \nonumber \\ P_{RD}({\textbf {T}}^+)= &  \epsilon _R P_{RD}({\textbf {T}}^-). \end{aligned}$$

Because domestic and wild salmon hosts are at equilibrium, $F_D^*$ and $F_W^*$ are constants. We numerically simulate the model using the *deSolve* package (Soetaert et al. 2010) in R version 4.3.3 (R Core Team 2024).

We use generic parameter values to capture emergent patterns related to the evolution, conservation, and economic outcomes of interest (Table 2, Generic values). The generic values parallel (Murray 2011). We also provide model outcomes for more realistic parameters, drawing on past empirical and theoretical work in the sea louse-salmon aquaculture system for system-specific parameter values (Table 2, System-specific values); this parallels the parameter choices in Bateman et al. (2020). We simulate evolution using an invasion analysis. We first run simulations with a susceptible-only population for a long enough period for it to equilibrate (1825 time steps), then we replace a small amount of susceptible lice in the farm environment ($P_{SD} = 0.001$) with resistant, and allowed invasion for the equivalent period as the burn-in time. For the generic case, the time step does not have a particular unit. For the system-specific case, the unit of time is days and the total simulation time is ten years, approximately the period in which resistance developed in BC, Canada (Godwin et al. 2022).

### Model analysis

We investigate metrics for evolutionary, conservation, and economic outcomes across the final 30 time steps (generic case) or 90 days (system-specific case). These time periods were selected because they allow the model to reach a steady state of recurrent cycles, and include a sufficient number of treatments to understand differences between alternative treatment thresholds and efficacies (ranging from zero to $>500$ treatments over 30 time steps for the generic parameters, and zero to $\sim$30 treatments over 90 days for the system-specific parameters). For the evolutionary outcome, we take the final proportion of resistant lice in the domestic environment ($\frac{P_{RD}}{P_{RD} + P_{SD}}$), averaged across the final time steps. For the conservation outcome, we take the final juvenile wild salmon population size ($F_J$) for each time step, averaged across the final time steps of the simulation. For the economic outcome, we estimate total economic losses (*C*) based on cost of treatment ($V_T$), the number of treatments ($Q_T$), the average daily parasite burden in the domestic environment ($\frac{P_{DR} + P_{DS}}{F_D}$) and loss of profits from parasite-induced decreased quality ($V_P$) during the final time steps of the simulation (Murray 2011):

12 $$\begin{aligned} C = Q_T V_T + \displaystyle \frac{P_{DR} + P_{DS}}{F_D} V_P. \end{aligned}$$

While this method ignores potential non-linearities in costs related to louse burdens, production time, and treatment application (Kragesteen et al. 2019), it captures the positive relationships between louse burdens, treatment application, and profit losses (Murray 2011). Parameters for economic losses are not drawn from the literature, but rather adjusted so that some treatment is more economically favorable, compared to no treatment (following Murray , 2011). We re-scale the conservation and economic model outcomes to be between 0 and 1 by dividing each output value by its maximum across all simulated scenarios.

To understand the trade-offs between economic, conservation, and evolutionary outcomes, we use Pareto efficiency frontiers (reviewed in Lester et al., 2013). Efficiency frontiers are useful because they directly compare outcomes in their natural units, rather than requiring a complex transformation of non-economic outcomes to economic values. To create each efficiency frontier, we plot model outcomes from various management simulations using generic parameters against each other. For example, to create the economic-conservation frontier, the conservation outcome (out-migrating juvenile salmon population size) is directly plotted against the economic outcome (louse-related losses with high treatment cost), where each point is a unique management simulation (treatment threshold/treatment efficacy combination). We compare the out-migrating juvenile salmon population size to the economic losses directly, rather than converting both outcomes into dollar values. We consider low economic losses, high juvenile salmon population sizes, and low resistance to be positive outcomes, i.e., we negate economic losses and proportion of resistant lice when plotting efficiency frontiers. The frontier is then the outermost edge of the plotted points. The frontier gives the maximized value of one outcome, given the other for the specific management levers in place, and vice versa. The shape of the frontier identifies how strongly the two outcomes tradeoff, with convex frontiers suggesting little-to-no trade-off between the outcomes, linear frontiers suggesting direct trade-offs between the outcomes, and concave frontiers suggesting a strong trade-off between the outcomes (Lester et al. 2013).

**Fig. 2:**
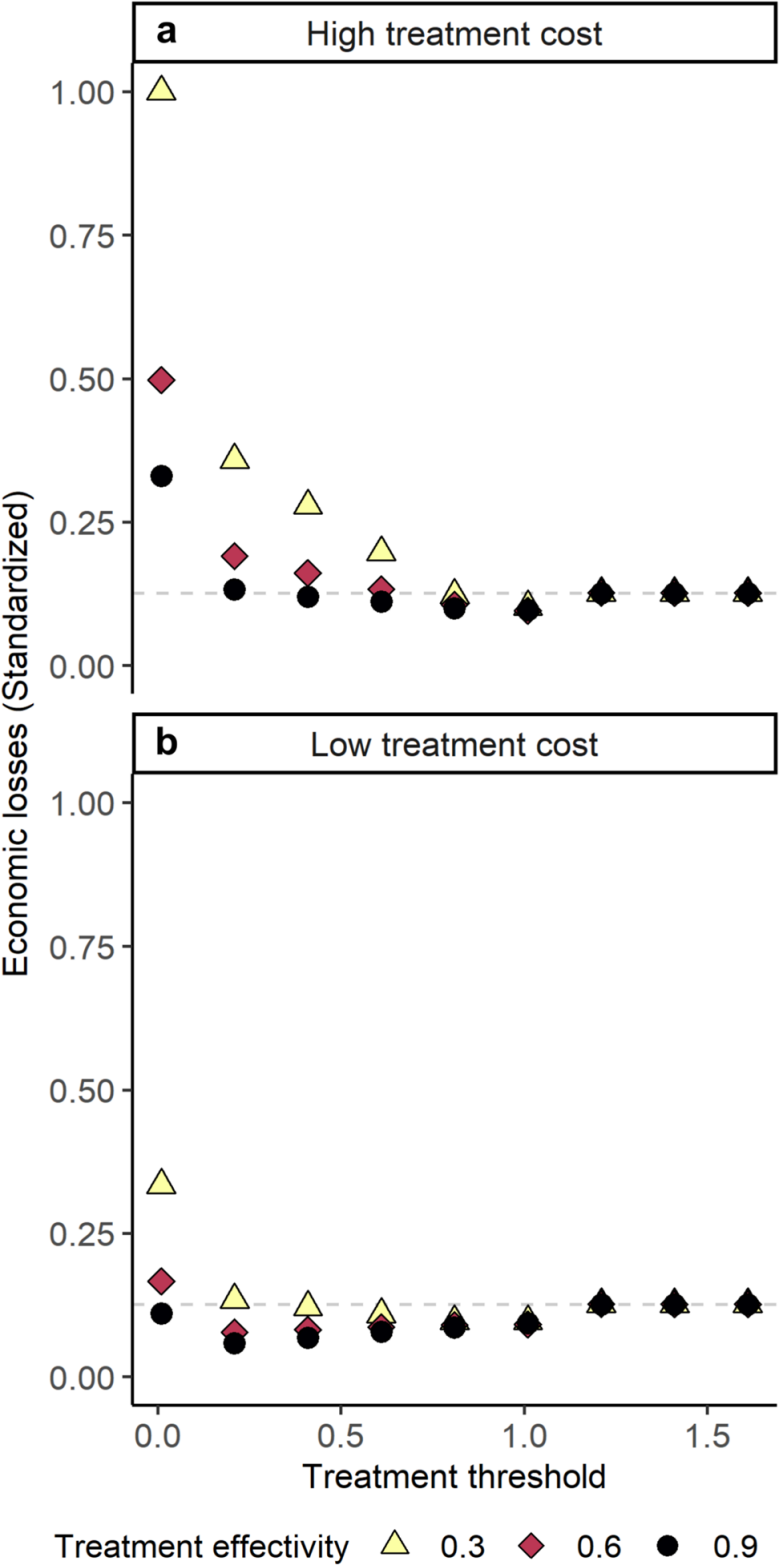
Economic outcomes. Treatment threshold (*x* axis, *M*) and treatment efficacy to susceptible lice (point color and shape, $\epsilon _S$) affect economic outcomes following Eq. 12 by determining the number of treatments and number of lice, on average, across the last 30 time steps of the simulation period. Economic losses (*y* axis) are resealed to be between 0 and 1 by dividing by the maximum economic loss calculated across all scenarios. Dashed light gray line gives loss without any treatment (i.e., maximum cost of louse burden). Panels show high (**a**) and low (**b**) cost of treatment

## Results

### Economic outcomes

As long as treatment expense is moderate and treatment is effective, occasional treatment (intermediate treatment threshold *M*) leads to the lowest economic losses (Fig. 2). Compared to never treating, occasional treatment application helps reduce the average louse burden in the farm environment (reducing output losses due to louse burdens, $\frac{P_{DR} + P_{DS}}{F_D} V_P$), without greatly increasing the costs associated with treating frequently ($Q_T V_T$). As treatment becomes cheaper relative to losses associated with louse burdens (Fig. 2b), more frequent treatment (lower treatment threshold *M*) reduces economic losses, so long as treatment remains effective. In some cases, even near continuous treatment (i.e., high-dose refuge strategy, $M \rightarrow 0$) can be more economically favorable than never treating if treatment has a very low cost relative to the loss associated with louse burdens. However, for treatments that are very ineffective (light yellow triangles, $\epsilon _S = 0.3$), frequent treatment increases economic losses.

**Fig. 3:**
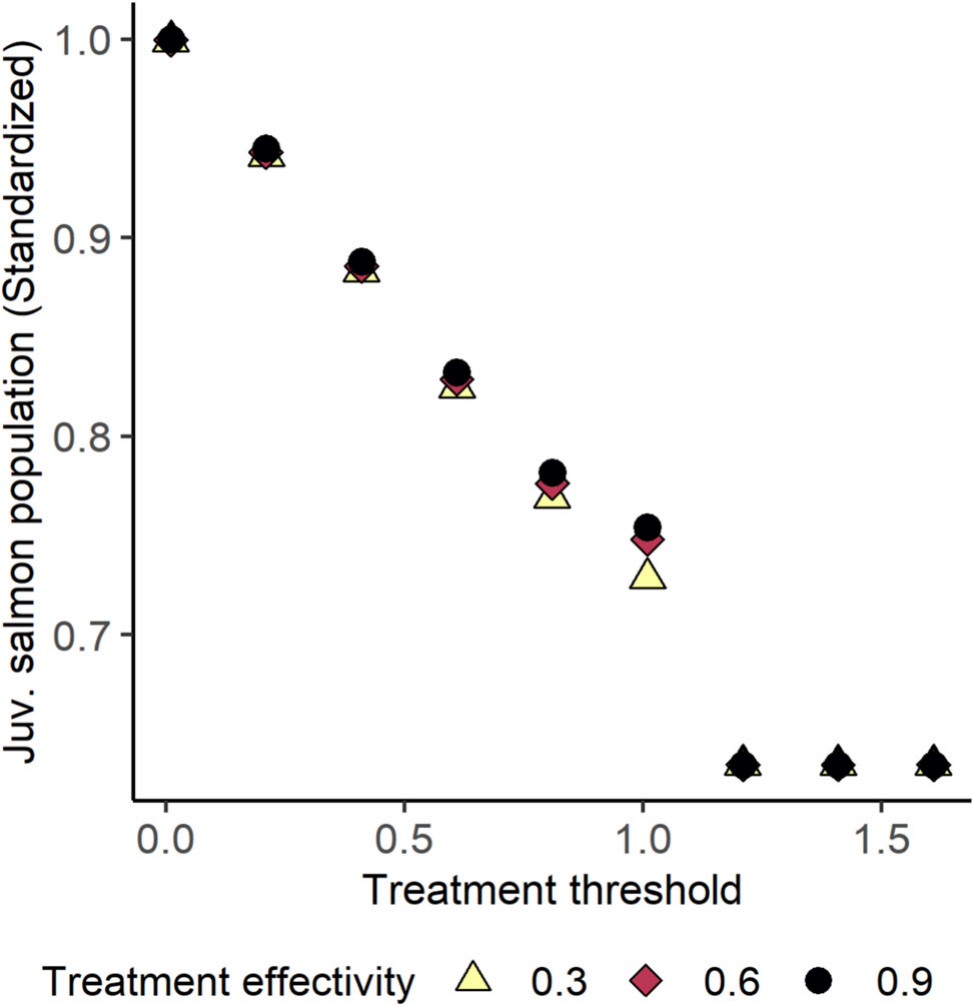
Conservation outcomes. Treatment threshold (*x* axis, *M*) and treatment efficacy to susceptible lice (point color and shape, $\epsilon _S$) affect the number of juvenile salmon hosts in the link environment by influencing the number of lice that move from the domestic to link environment. Out-migrating wild juvenile salmon population size (*y* axis) is averaged across the last 30 time steps of the simulation period and resealed to be between 0 and 1 by dividing by the maximum number of juvenile salmon hosts in the link environment calculated across all scenarios

**Fig. 4:**
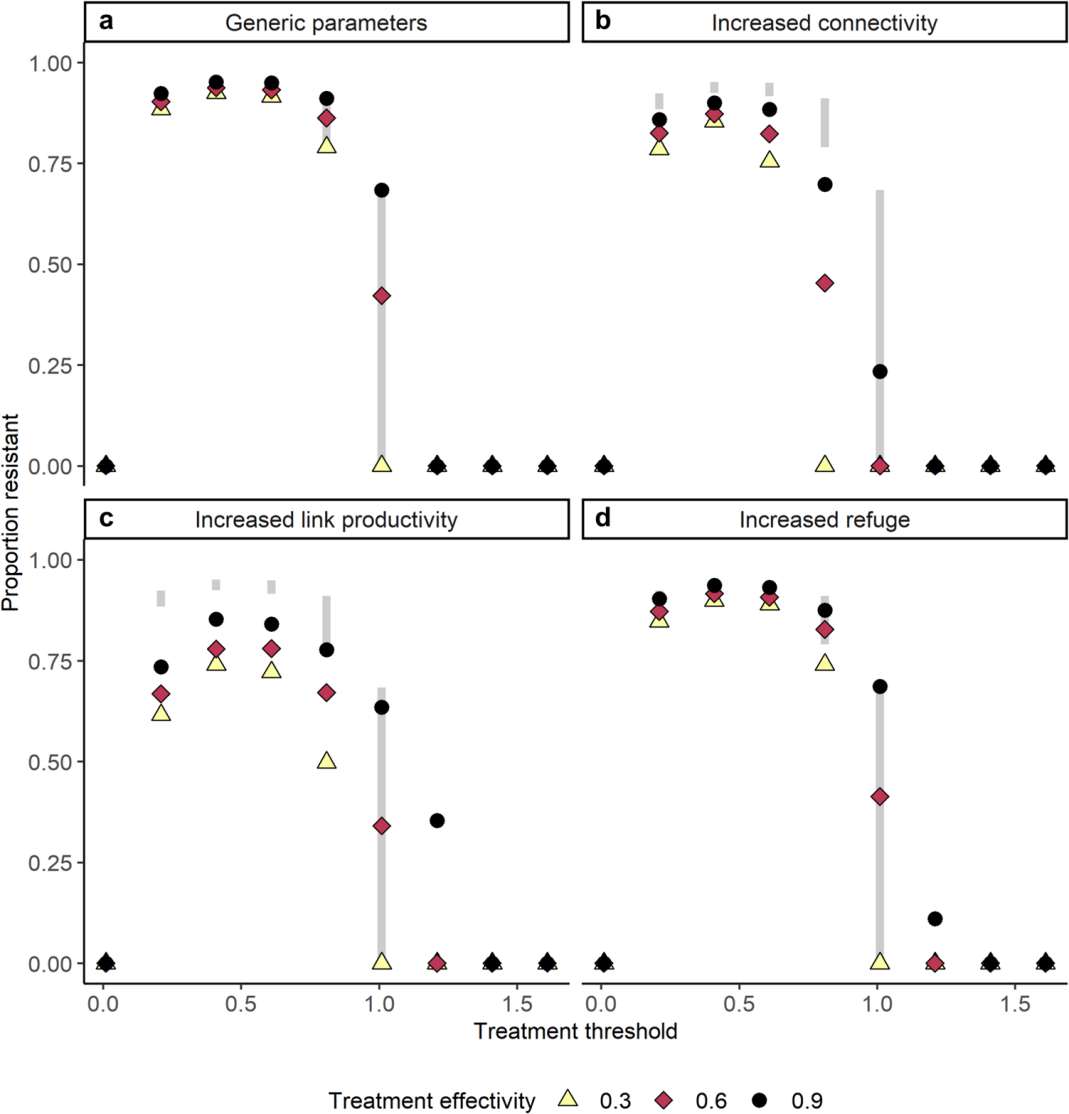
Evolutionary outcomes. Treatment threshold (*x* axis, *M*) and treatment efficacy to susceptible lice (point color and shape, $\epsilon _S$) affect evolutionary outcomes by determining the selection strength of parasite treatments. Light gray bars are a visual aid to compare resistance proportion (*y* axis) of the base parameter case to other parameter cases. Panels show base generic parameters (**a**), increased connectivity ($\phi _{LD} = \phi _{DL} = 0.15$, **b**), increased link population productivity ($\eta _{N1} = 1.25$, **c**) and increased wild refuge population ($F_W = 20$, **d**)

### Conservation outcomes

High treatment efficacy and low treatment threshold both increased the out-migrating juvenile salmon population size (Fig. 3). Mechanistically, both of these treatment strategies decrease the louse burden in the farm environment on average, decreasing the number of lice that spill back from the farm population to the out-migrating juvenile salmon population. As a result, louse burdens on out-migrating juvenile salmon decrease, leading to lower mortality and increased population size. This result comes directly from the model construction: the only source of lice infecting out-migrating juvenile salmon is from the domestic environment. When there is no spillback ($\phi _{LD} = 0$), out-migrating juvenile salmon population sizes are maximized.

### Evolutionary outcomes

Resistance evolution slowed for either (a) strategies that treated frequently and were coupled with high gene flow, or (b) strategies that imposed little selective pressure, matching with past work (Murray 2011; Ashander 2010; Stratonovitch et al. 2014; McEwan et al. 2015; Kreitzman et al. 2017; Bateman et al. 2020; Fig. 4). We reproduced the low selection strategy from Murray (2011) through either increasing treatment threshold (Fig. 4, right most points in each panel) or decreasing the treatment efficacy to susceptible lice (Fig. 4, light yellow triangles, $\epsilon _S = 0.3$). Moderate treatment strategies (i.e., moderate treatment thresholds) lead to high levels of resistance evolution. Additional management efforts such as increasing connectivity (Fig. 4b), increasing link population productivity (Fig. 4c), or increasing the refuge population size (Fig. 4d) slowed resistance evolution compared to the base parameter case (Fig. 4a and light gray bars; parameters as in Table 2, generic values).

We reproduced the high-dose refuge effect of McEwan et al. (2015) and Bateman et al. (2020) (Fig. 4, $M \rightarrow 0$). This strategy depends on near-constant treatment, i.e., near zero treatment thresholds and in some cases, the effect was lost completely if there was insufficient gene flow. Increasing conservation of the link (increasing $\eta _{N1}$) and/or wild population ($F_W^*$), or increasing the connectivity across the link and domestic environment ($\phi _{DL}, \phi _{LD}$) restored the high-dose refuge effect, but generally only for cases where treatment was nearly constant. In particular, as the amount of spillback from the farm to juvenile salmon ($\phi _{LD}$) decreased while the amount of spillover from spawning to farm salmon ($\phi _{DL}$) remains constant or increases, resistance evolution slows because there are relatively more susceptible lice per host returning during spawning. This result specifically comes from the three-environment structure, which more closely matches the Pacific salmon life cycle: because the spillover of lice is directional, off-farm selection against resistant lice is stronger in the three-environment model (paralleling structure of Bateman et al. (2020)) than the two-environment model (paralleling structure of Murray (2011)).

**Fig. 5:**
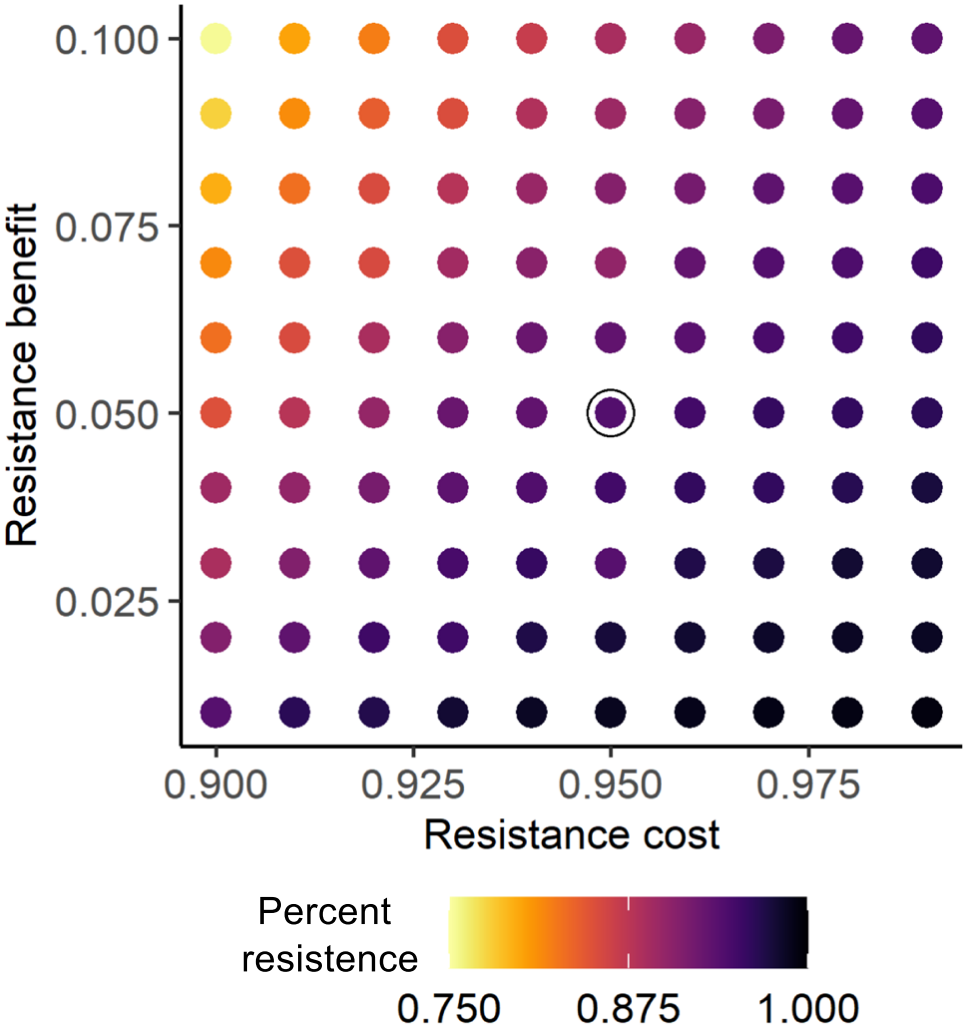
Biological trade-offs of resistance. The cost of resistance ($\frac{\lambda _R}{\lambda _S}$, *x* axis) and the relative selective efficacy ($\gamma$, resistance benefit, *y* axis) determine the relative proportion of lice that are resistant at the end of a simulation, when treatment threshold and treatment efficacy to susceptible lice is constant ($M = 0.7$ and $\epsilon _S = 0.9$, respectively). The color of the points gives the percent of resistant lice at the end of the simulation, averaged over the last 30 days of the simulation. The circled point shows the resistance cost and benefit modeled using generic parameters ($\frac{\lambda _R}{\lambda _S} = 0.95; \gamma = 0.05$)

In response to treatment threshold, resistance evolution shows a sharp, threshold-like behavior. Using extreme (near zero or very high) treatment thresholds tended to decrease the amount of resistance evolution. Reducing treatment efficacy to susceptible lice ($\epsilon _S$, where treatment efficacy to resistant lice is $\gamma \epsilon _S$, $0< \gamma < 1$) decreased resistance evolution for a given treatment threshold, because the benefit of resistance evolution (decreased mortality) was lower for the same cost (Fig. 5).

### Efficiency frontiers

**Fig. 6:**
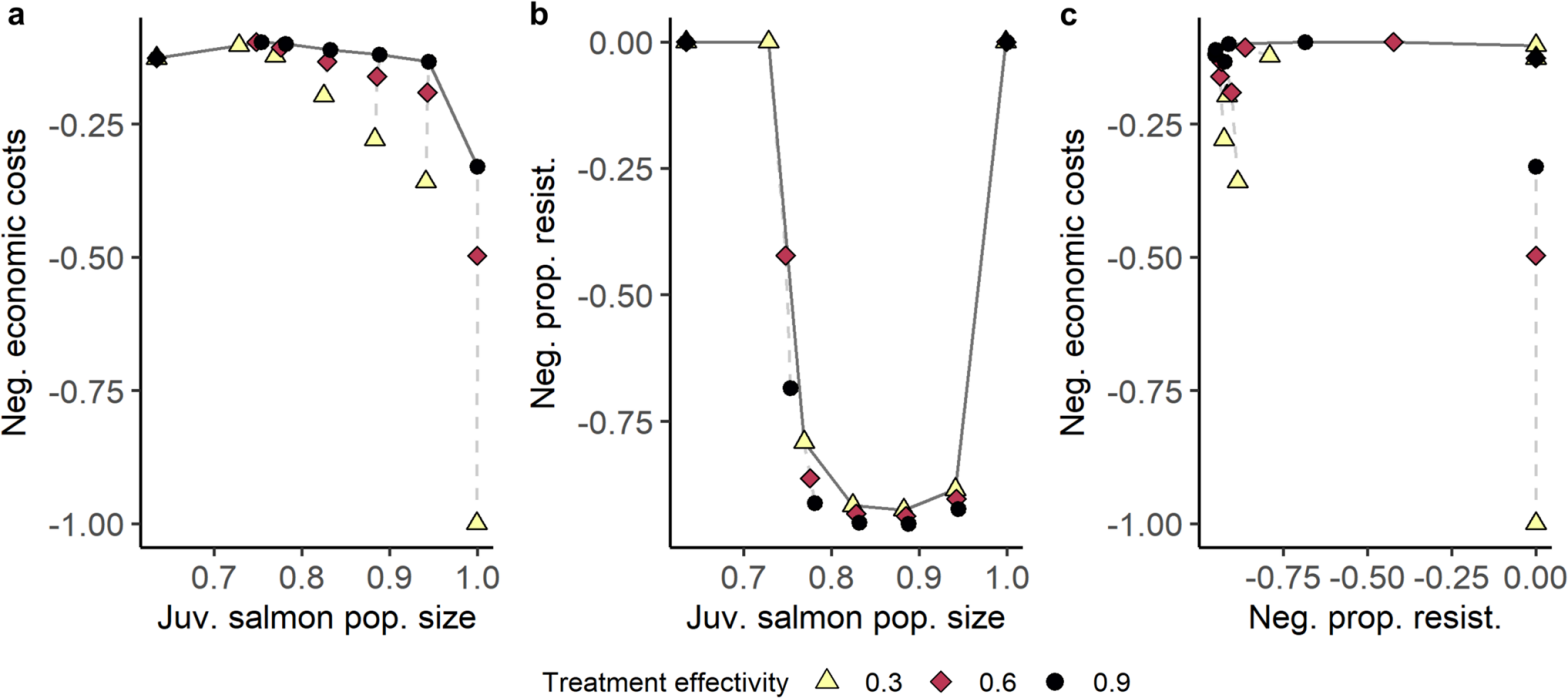
Two-dimensional efficiency frontiers. Plots give pairwise efficiency frontiers, solid dark gray line, across all three outcomes of interest. Efficiency frontiers give the jointly maximized value for pairs of outcomes on their natural scales. Two-dimensional frontiers are **a** economic (*y* axis)-conservation (*x* axis), **b** evolutionary (*y* axis)-conservation (*x* axis), and **c** economic (*y* axis)-evolutionary (*x* axis). Point color gives treatment efficacy to susceptible lice ($\epsilon _S$), and dashed lines between points indicate shared treatment threshold (*M*) to show relationships when holding either treatment efficacy or threshold constant. All outcomes are simulated from generic parameters; the economic outcome is calculated using the high treatment cost case

Trade-offs between economics and conservation outcomes were weak (Fig. 6a, concave) across all model scenarios, indicating potential win-win outcomes managers could achieve via disease management choices. In other words, there are treatment efficacy and threshold choices for disease management that allow both relatively low economic losses and relatively high juvenile wild salmon populations to persist. Across all model scenarios, management outcomes are jointly maximized when treatment efficacy was high (Fig. 6, dark circles, $\epsilon _S = 0.9$). As treatment efficacy to susceptible lice decreased ($\epsilon _S$), trade-offs became stronger and more direct, with the frontier shifting from concave to linear (Fig. 6a). This represents a shift from weak trade-offs to more direct trade-offs because, with a linear frontier, changes to the value one outcome simultaneously change the value of the other outcome (Lester et al. 2013).

Trade-offs between conservation and evolution show non-monotonic frontiers across all model scenarios (Fig. 6b), which were jointly maximized when treatment efficacy was low (Fig. 6, light yellow triangles, $\epsilon _S = 0.3$), because this decreases resistance evolution more strongly than it decreases the out-migrating juvenile salmon population size. Frontiers across particular treatment efficacies are similarly shaped, while frontiers across particular treatment thresholds (Fig. 6, along dashed lines) are more direct. When there is no treatment, and thus no selection, there was low treatment resistance and high juvenile salmon populations. As the treatment threshold increases, juvenile salmon populations and treatment resistance increase. However, when juvenile salmon populations are maximized, there is less evolution of resistance. This highlights that while resistance and conservation can show synergies, increased link population size does not necessarily reflect less resistance evolution. This is because the treatment threshold is the main determinant of both evolutionary and conservation outcomes as measured here. Instead of considering increased link population size as the direct mechanism relating to resistance evolution, our model demonstrated that (a) increases in link population growth rate and (b) decreased spillback and (c) increased spillover are factors that alter evolutionary outcomes by increasing gene flow.

Trade-offs between economics and evolution are weak across all model scenarios (Fig. 6c). Near or optimal outcomes across both economic and evolutionary dimensions are possible at the expense of the link population size. The upper rightmost point on the economic-evolution frontier (Fig. 6c) corresponds to never treating sea lice, leading to high domestic louse burdens and low link population sizes. Frontiers across particular treatment efficacies (Fig. 6, point color) are scattered and non-monotonic. Frontiers across particular treatment thresholds (Fig. 6, along dashed lines) show weak trade-offs that were more relaxed as the treatment threshold increased. If lice are particularly costly ($$ C_Q>> C_T) $$, the trade-off is stronger, because not treating becomes less desirable (Figs. 2 and 6).

### Alternative, system-specific parameterization

**Fig. 7:**
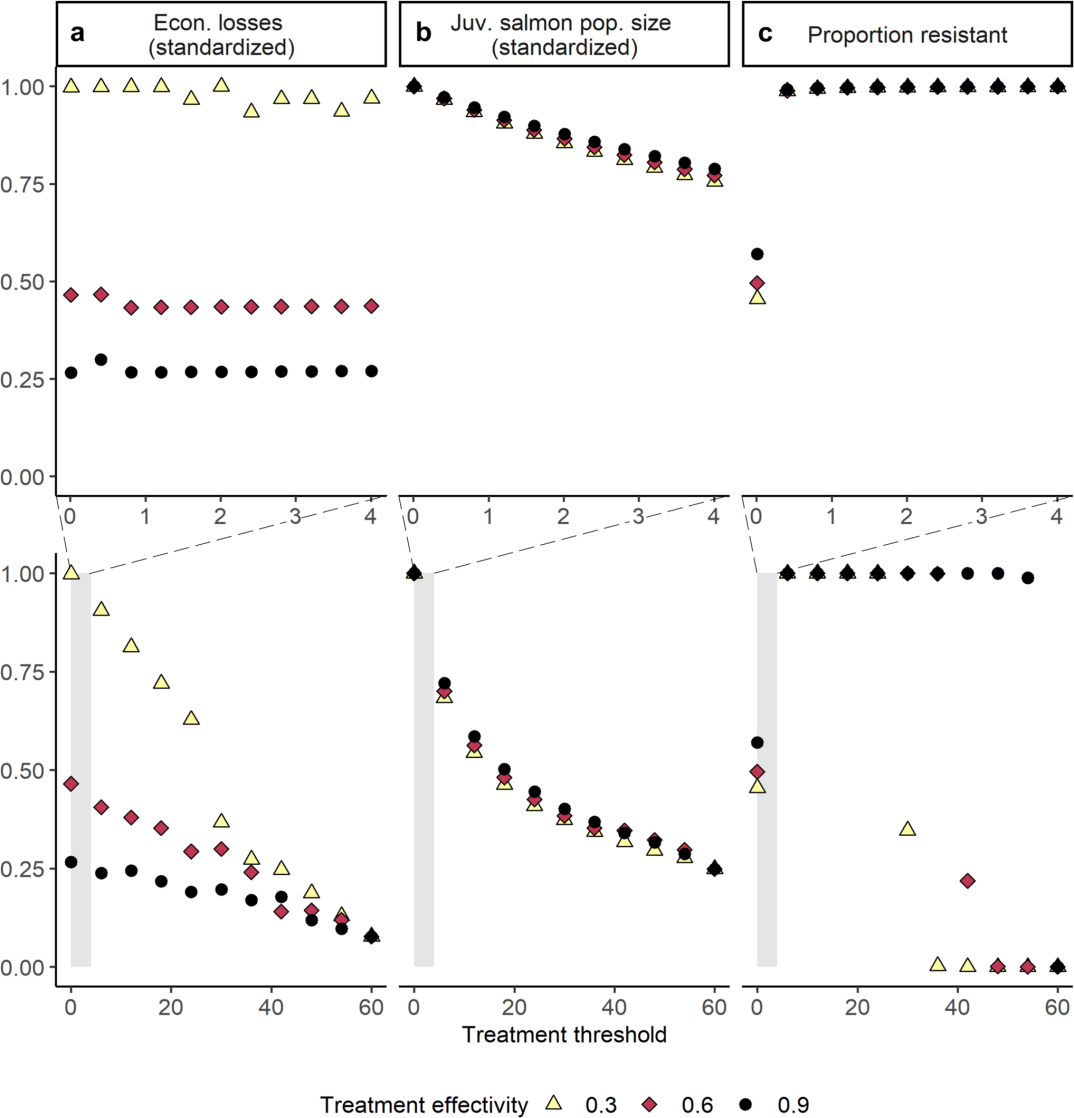
Simulated outcomes from system-specific sea louse-salmon aquaculture system (BC) parameter values. Varying treatment threshold (*x* axis) and treatment efficacy to susceptible lice (point color) lead to similar patterns in outcomes as generic parameters. The upper panels give a subset of the treatment thresholds in the lower panels, in order to visualize the outcomes across a realistic range of treatment thresholds used in practice globally. We re-scale economic and conservation outcomes to be between 0 and 1 by dividing each output value by its maximum across all simulated scenarios; economic outcomes are less smooth compared to the generic parameter value case (Fig. 2) because treatment is relatively infrequent in linear time in comparison. Parameters as listed in Table 2 under “System-specific values; outputs” are averaged over the final 3 months of the simulation following resistant lice invasion to capture multiple treatment cycles

We primarily focused on using generic parameters to capture patterns and understand mechanisms in the system, rather than empirically-derived parameters. Using more empirically realistic parameters (Frazer et al., 2012; Peacock et al., 2014; Bateman et al., 2020; Table 2 System-specific values), we saw similar patterns with the system-specific values compared to the generic base case (Fig. 7; note outcomes averaged over the final 3-month period of the simulation).

## Discussion

Using a stylized model to describe the sea louse-salmon aquaculture system, we observed the capacity for discrete, threshold-based treatment applications to moderate evolutionary, conservation, and economic outcomes. We found that moderate treatment thresholds, similar to those currently used in many salmon aquaculture systems (Krkošek 2010b; Jeong et al. 2023), lead to high levels of resistance evolution, even in the presence of large refuge louse populations (Fig. 4). Our model showed that economic and conservation win-wins are possible: there are treatment threshold choices that lead to relatively high out-migrating juvenile wild salmon population sizes and relatively low economic losses, especially when treatment is very effective or treatment is cheap (Figs. 2b and 6a). However, positive evolutionary outcomes are harder to capture and occur more often when treatment efficacy is low (Figs. 4 and 6b). Capturing positive economic, evolutionary, and conservation outcomes might require management action beyond the regulation of louse treatment threshold and minimum treatment efficacy.

### Threshold-based treatment moderates outcomes

The high-dose refuge approach is weakened by the introduction of discrete, threshold-based treatment application, but could be preserved with efforts to increase gene flow. Here, our model paralleled the results of past models, which suggest that the high-dose refuge effect only occurs under intensive treatment (Murray 2011) and random mating (Campagne et al. 2015). Similarly to past work, increasing connectivity between the domestic and link environments, or increasing conservation effort through increased link population productivity or increased wild refuge size helped strengthen the high-dose refuge effect (Ashander 2010; McEwan et al. 2015; Kreitzman et al. 2017; Bateman et al. 2020; Fig. 4). This result comes from the model structure: because lice move directional from the production environment to the wild environment via the link environments, resistant lice face strong negative selection in three environments before returning to the domestic environment. Efforts to increase connectivity or link population productivity, then, help increase the relative gene flow a production environment receives, increasing the high-dose refuge effect (McEwan et al. 2015; Bateman et al. 2020).

The decomposition of adult louse treatment into treatment efficacy and treatment threshold moderated model outcomes. This decomposition led to two distinct mechanisms that can create low selection approaches: both lower treatment efficacy (low $\epsilon _S$) or rare treatment (high *M*) led to slowed resistance evolution (Fig. 4). Generally, the treatment threshold has a stronger effect on resistance evolution, but for a given treatment threshold, lower treatment efficacy helps slow resistance evolution. This contrasts (Coates et al. 2023), where decreased efficacy lead to increased number of treatments increasing selection, likely because our analysis focuses two competing phenotypes (invasion approach), rather than population genetic approaches and continuous phenotypes utilized by Coates et al. (2023).

Greater treatment efficacy decreased economic losses (Fig. 2; Kragesteen et al., 2019). Increased treatment efficacy could result from increased dosage or effort in an existing treatment, or development of new, more effective treatments. For high treatment efficacy, frequent treatment becomes more economically favorable when treatment is relatively cheap. However, as treatment efficacy decreases and treatment is relatively expensive, low-frequent treatment and in some cases, never treating, becomes more economically favorable. Combined, this suggests that considering both dimensions of treatment can help better minimize the negative downstream impacts of disease (Groner et al. 2016; Baker et al. 2018; Barrett et al. 2020; Jeong et al. 2021; Godwin et al. 2021a; Coates et al. 2023).

### Model limitations

Our model aims to capture components of the sea louse-salmon system relevant to evaluating a discrete, threshold-based treatment application, but inevitably has several key assumptions. Our model represents a case with an isolated farm or a set of synchronized farms (i.e., all in a region have identical dynamics) in the domestic environment and did not consider the movement of louse across farm networks that might change gene flow, treatment dynamics, and model outcomes (Peacock et al. 2016; Kragesteen et al. 2019). Similarly, wild salmon systems show high seasonality in migratory patterns, so spillover and spillback only occur for part of the year. Both of these factors likely create further limitations to the high-dose refuge effect and increase resistance evolution (Campagne et al. 2015) while decreasing wild salmon populations (Godwin et al. 2021a).

We made several simplifying assumptions about louse biology. First, we assumed that each louse morph distributes following independent, identical, Poisson distributions. While this assumption is mathematically convenient, sea lice tend to be more clustered in their distribution, i.e., overdispersed (Revie et al. 2005). Our assumption of independent, identical, Poisson morph distribution might overestimate the negative impact of lice to juvenile salmon populations, which could further ease the trade-off between on-farm economic outcomes and conservation outcomes. Second, we ignored seasonality. Environmental forcing can significantly limit or drive sea louse reproduction (Jansen et al. 2012; McEwan et al. 2015; Godwin et al. 2021a). Adding seasonality to our model could improve the strength of the high-dose refuge effect because most louse reproduction happens in warmer, summer months, when wild and domestic lice exchange (Campagne et al. 2015) and lead to more dramatic effects on wild juvenile salmon. Moreover, alternate reservoir hosts (Godwin et al. 2021a) could complicate lice exchange between farm and wild environments by providing an additional population of susceptible lice and increasing connectivity between farm and wild environments.

By using invasion analysis, we modeled resistance as a fixed phenotype and lice as clonal, rather than modeling the complete genetic architecture or allowing resistance to evolve as a continuous trait. Because our model consisted only of invasion analysis, we did not find the joint resistance cost-benefit that would be optimized to given particular treatment scenarios; instead, the model reflects the three outputs of interest given a single invasion of a new, fixed-resistance gene among lice. If resistance is a recessive trait or continuous trait, gene flow could further slow resistance evolution (Coates et al. 2023). This could be particularly important to help prolong the high-dose refuge effect (Campagne et al. 2015); thus, our model might overemphasize the erosion of the high-dose refuge effect by discrete treatment.

We provide two parameter cases as explorations of the model. However, some of our parameter estimates are likely unrealistic and might overestimate the trade-offs associated with resistance phenotypes, which empirical work suggests may be minimal (reviewed in Coates et al., 2021). Less intensive trade-offs led to more resistance evolution (Fig. 5), unless lice are never treated. We include results across multiple parameter values that could be of interest to demonstrate the robustness of various findings (Figs. 2, 4, 5, 7), e.g., various amounts of gene flow between environments, various treatment costs for economic analysis, various resistance cost levels. Thus, we feel our model captures the qualitative patterns that result from various treatment thresholds and efficacies, while recognizing that our model is not quantitatively predictive in nature.

### Management implications

By modeling discrete, threshold-based treatment, we demonstrated that resistance evolution can evolve either when using moderate treatment thresholds or when gene flow between wild and domestic parasite populations is low (Fig. 4). Moderate treatment thresholds, relative to louse production, aiming to balance economic and conservation outcomes, are commonly imposed by regulatory bodies (Jansen et al. 2012; Jeong et al. 2023). For example, the Department of Fisheries and Oceans (Canada) presently requires BC salmon farms to treat sea lice at a threshold of three motile lice per fish (Krkošek 2010b). Moreover, aquaculture producers might treat at moderate thresholds, regardless of policy directives, to preserve economic viability (Kragesteen et al. 2019; Godwin et al. 2021b; Jeong et al. 2023). In our model, the choice to use moderate thresholds, either through regulation or to minimize economic losses, leads to resistance evolution, even when there are large refuge populations. This matches with past work on the high-dose refuge effect, because moderate thresholds violate the ecological assumptions needed to establish the effect (Ives and Andow 2002). In addition, it aligns with observations that sea lice have largely become resistant to chemical treatments across the globe (Aaen et al. 2015; Coates et al. 2021). The high levels of resistance evolution, partially attributable to the use of moderate treatment thresholds in our model, has forced farms to use more expensive and less effective, non-chemical treatments (Overton et al. 2019; Barrett et al. 2020; Boerlage et al. 2024).

Our model is non-specific to treatment type and represents treatment resistance broadly. The generality of our model represents any treatment that is (1) applied at a pre-specified threshold, (2) has some given efficacy, and (3) for which parasites can evolve resistance to Coates et al. (2021). In other words, our results both describe past evolution to chemical treatments, such as emamectin benzoate, and can also describe evolution to emerging chemical and non-chemical treatments, such as mechanical delousing. In our model, the high-dose refuge effect strategy only occurred with near continuous treatment and resulted in positive conservation and evolution outcomes. In contrast, the low selection strategy resulted in positive evolution outcomes and positive economic outcomes (when treatment was very expensive). To better slow resistance evolution, our model suggests the use of additional management levers beyond discrete treatment. For example, conservation of refuge wild fish populations (e.g., increasing wild salmon productivity ($\eta _{N1}$) and/or wild population size ($F_W^*$)) and manipulating connectivity ($\phi _{DL}, \phi _{LD}$) between production and wild environments (e.g., careful farm placement and regulated treatment timing) can help preserve the high-dose refuge effect, protect sympatric, wild populations and reduce economic impacts of disease (Fig. 4; Peacock et al., 2013; Baker et al., 2018; Barrett et al., 2020; Jeong et al., 2021; Godwin et al., 2021a; Coates et al., 2023).

Our model indicates that capturing positive management outcomes across evolutionary, conservation, and economic perspectives is not possible through only the choice of treatment threshold and treatment efficacy (Fig. 6). On our efficiency frontiers, economic and conservation win-wins occur when treatment was highly effective, while conservation and resistance outcomes are jointly maximized when treatment efficacy was low. Economic and conservation trade-offs can be further eased by lowering the cost of treatment. Use of additional management levers, such as wild fish conservation, treatment timing, and farm placement, might allow managers to capture more net benefits across these three perspectives and should be considered in future work.

## Data Availability

Model code is available on GitHub under the repository lauriebalstad/disc-treat_eco-evo-econ, found at https://github.com/lauriebalstad/disc-treat_eco-evo-econ.
